# (4-Nitro­phenolato)(subphthalo­cyaninato)boron(III)^1^

**DOI:** 10.1107/S1600536810050580

**Published:** 2010-12-08

**Authors:** Andrew S. Paton, Alan J. Lough, Timothy P. Bender

**Affiliations:** a Department of Chemical Engineering & Applied Chemistry, University of Toronto, 200 College Street, Toronto, Ontario, Canada M5S 3E5; b Department of Chemistry, University of Toronto, 80 St. George Street, Toronto, Ontario, Canada M5S 3H6

## Abstract

The main feature of the structure of the title compound, C_30_H_16_BN_7_O_3_ or NO_2_PhO-BsubPc, are pairs of mol­ecules linked through π-inter­actions between the concave faces of the BsubPc fragments at a distance of 3.5430 (11) Å across an inversion centre. However, the angle between the planes of the five- and six-menbered rings involved in this inter­action is 1.44 (10)°, causing the inter­acting BsubPcs units to be slightly askew rather than parallel as is typical for π-stacking inter­actions.

## Related literature

For a general review of boronsubphthalocyanine compounds (BsubPcs), see: Claessens *et al.* (2002 ▶). For synthesis of BsubPcs and their derivatives, see: Zyskowski & Kennedy (2000 ▶); Claessens *et al.* (2003 ▶); Paton *et al.* (2010 ▶). For the application of BsubPcs in organic electronic devices, see: Morse *et al.* (2010 ▶) and references cited therein; Gommans *et al.* (2007 ▶). For related structures of non-halogenated BsubPc derivatives, see: Potz *et al.* (2000 ▶); Paton *et al.* (2010*a* ▶,*b* ▶).
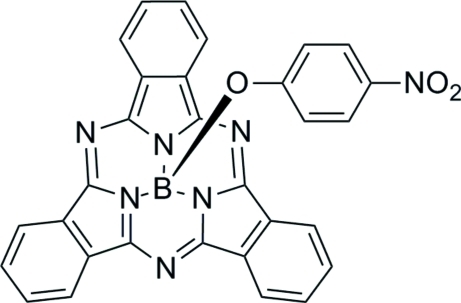


## Experimental

### 

#### Crystal data

C_30_H_16_BN_7_O_3_
*M* *_r_* = 533.31
Monoclinic, 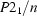

*a* = 15.6597 (4) Å
*b* = 8.2959 (1) Å
*c* = 19.5409 (5) Å
β = 110.3060 (9)°
*V* = 2380.82 (9) Å^3^
*Z* = 4
Mo *K*α radiation
μ = 0.10 mm^−1^
*T* = 150 K
0.40 × 0.26 × 0.20 mm

#### Data collection

Nonius KappaCCD diffractometer
Absorption correction: multi-scan (*SORTAV*; Blessing, 1995 ▶) *T*_min_ = 0.786, *T*_max_ = 1.000
19982 measured reflections
5413 independent reflections
3646 reflections with *I* > 2σ(*I*)
*R*_int_ = 0.052

#### Refinement

*R*[*F* ^2^ > 2σ(*F* ^2^)] = 0.049
*wR*(*F* ^2^) = 0.141
*S* = 1.03
5413 reflections
371 parameters
H-atom parameters constrained
Δρ_max_ = 0.27 e Å^−3^
Δρ_min_ = −0.33 e Å^−3^

### 

Data collection: *COLLECT* (Nonius, 2002 ▶); cell refinement: *DENZO-SMN* (Otwinowski & Minor, 1997 ▶); data reduction: *DENZO-SMN*; program(s) used to solve structure: *SIR92* (Altomare *et al.*, 1994 ▶); program(s) used to refine structure: *SHELXTL* (Sheldrick, 2008 ▶); molecular graphics: *PLATON* (Spek, 2009 ▶); software used to prepare material for publication: *SHELXTL*.

## Supplementary Material

Crystal structure: contains datablocks global, I. DOI: 10.1107/S1600536810050580/nc2204sup1.cif

Structure factors: contains datablocks I. DOI: 10.1107/S1600536810050580/nc2204Isup2.hkl

Additional supplementary materials:  crystallographic information; 3D view; checkCIF report

## Figures and Tables

**Fig. 1.**
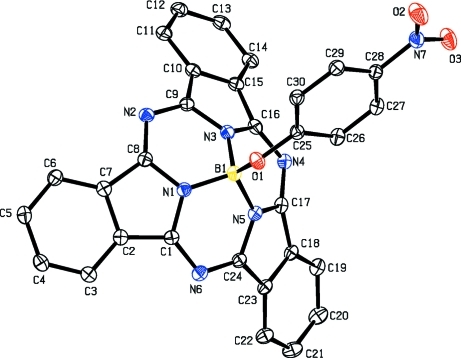
The molecular structure with labels of NO2PhO-BsubPc with displacement ellipsoids drawn at the 30% probability level.

**Fig. 2.**
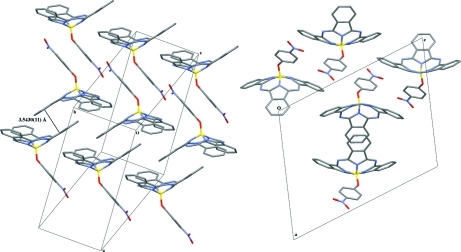
Extended crystal structure of NO2PhO-BsubPc shown from two views.

## supplementary crystallographic information

### Comment

We report the crystal structure of 4-nitrophenoxy-boronsubphthalocyanine (NO_2_PhO-BsubPcs), which possesses an electron withdrawing group in the *para* position of the phenoxy molecular fragment. We have recently reported a study of the crystal structures of a series of *para*-substituted phenoxy-BsubPcs wherein most of the substituents were electron donating (alkyl, Paton *et al.*, 2010). Contained within the study was 4-fluorophenoxy-BsubPcs (FPhO-BsubPcs). While fluorine is moderately electron withdrawing we did not observe any difference in its crystal structure compared to the baseline phenoxy-BsubPcs. We have since reported the structure of a derivative with a stronger electron withdrawing group, 4-acetylphenoxy-BsubPcs. (Paton *et al.*, 2011) This structure was only slightly different from the typical FPhO-BsubPcs crystal packing motif. We synthesized the title compound as the next derivative in a series studying the effects of electron withdrawing groups on related compounds.

The title compound was prepared by a method described previously (Paton *et al.*, 2010; Claessens *et al.*, 2003), in which chloro-boronsubphthalocyanine (Cl-BsubPcs) is reacted with an excess of the appropriate phenol until substitution is complete. Further details are given in the experimental sections which accompany this article.

The molecular structure of the title compound obtained from benzene-heptane diffusion crystallization is shown in Fig. 1. The compound shows the expected bowl shape of the BsubPcs ligand. The boron-oxygen-carbon (B—O—C) angle in the molecule is 124.56 (14)°, which differs significantly from both the experimental (115.2 (2)°) and computational gas-phase (*ca* 115°) values of B—O—C angle for the typical phenoxy derivatized FPhO-BsubPcs (Paton *et al.*, 2010). Examining the torsion angle between the boron, oxygen, and the first two carbon atoms on the phenoxy substituent (B—O—C—C) gives values of -44.7 (3)°. In contrast, the angle associated with FPhO-BsubPcs is -91.0 (2)° relative to the plane of the BsubPcs fragment (Paton *et al.*, 2010).

The crystal structure of NO_2_PhO-BsubPcs (Fig. 2) shows pairs of BsubPcs fragments associated through a π-interaction separated by a centroid-to-centroid distance of 3.5430 (11) Å. These pairs of molecules form one-dimensional rows aligned with the *b* axis. The π-interaction creating the pairs is between two sets of BsubPcs fragments whose ring planes are not perfectly parallel; the planes of the two rings (C9/C10/C11/C12/C13/C14/C15 and C9/C10/C15/C16/N3 on neighbouring molecules) are at an angle of 1.44 (10)°.

### Experimental

Cl-BsubPc, synthesized by a procedure adapted from Zyskowski and Kennedy (2000), The title compound was synthesized using a method adapted from Claessens *et al.* (2003) and Paton *et al.* (2010): 4-Nitrophenoxy-boronsubphthalocyanine. Cl-BsubPc (0.510 g, 0.0012 mol) was mixed with 4-nitrophenol (0.567 g, 0.0041 mol) in toluene (10 ml) in a cylindrical vessel fitted with a reflux condenser and argon inlet. The mixture was stirred and heated at reflux under a constant pressure of argon for 17 h. Reaction was determined complete *via* HPLC by the absence of Cl-BsubPc. The solvent was evaporated under rotary evaporation. The crude product purified on a Kauffman column using standard basic alumina (300 mesh) as the adsorbent and dichloromethane as the eluent. The product elutes from the Kauffman column while the excess phenol remains adsorbed. The dichloromethane was then removed under reduced pressure yielding a dark pink/magenta powder of the title compound (0.223 g, 37%).

### Crystal data

**Table:**

C_30_H_16_BN_7_O_3_	*F*(000) = 1096
*M**_r_* = 533.31	*D*_x_ = 1.488 Mg m^−^^3^
Monoclinic, *P*2_1_/*n*	Mo *K*α radiation, λ = 0.71073 Å
Hall symbol: -P 2yn	Cell parameters from 19982 reflections
*a* = 15.6597 (4) Å	θ = 2.7–27.5°
*b* = 8.2959 (1) Å	µ = 0.10 mm^−^^1^
*c* = 19.5409 (5) Å	*T* = 150 K
β = 110.3060 (9)°	Needle, purple
*V* = 2380.82 (9) Å^3^	0.40 × 0.26 × 0.20 mm
*Z* = 4	

### Data collection

**Table:**

Nonius KappaCCD diffractometer	5413 independent reflections
Radiation source: fine-focus sealed tube	3646 reflections with *I* > 2σ(*I*)
graphite	*R*_int_ = 0.052
Detector resolution: 9 pixels mm^-1^	θ_max_ = 27.5°, θ_min_ = 2.7°
φ scans and ω scans with κ offsets	*h* = −20→20
Absorption correction: multi-scan (*SORTAV*; Blessing, 1995)	*k* = −10→10
*T*_min_ = 0.788, *T*_max_ = 1.002	*l* = −20→25
19982 measured reflections	

### Refinement

**Table:**

Refinement on *F*^2^	Secondary atom site location: difference Fourier map
Least-squares matrix: full	Hydrogen site location: inferred from neighbouring sites
*R*[*F*^2^ > 2σ(*F*^2^)] = 0.049	H-atom parameters constrained
*wR*(*F*^2^) = 0.141	*w* = 1/[σ^2^(*F*_o_^2^) + (0.0779*P*)^2^ + 0.3383*P*] where *P* = (*F*_o_^2^ + 2*F*_c_^2^)/3
*S* = 1.03	(Δ/σ)_max_ = 0.001
5413 reflections	Δρ_max_ = 0.27 e Å^−^^3^
371 parameters	Δρ_min_ = −0.33 e Å^−^^3^
0 restraints	Extinction correction: *SHELXTL* (Version 6.1; Sheldrick, 2008), Fc^*^=kFc[1+0.001xFc^2^λ^3^/sin(2θ)]^-1/4^
Primary atom site location: structure-invariant direct methods	Extinction coefficient: 0.0062 (13)

### Special details

**Table:**

Geometry. All e.s.d.'s (except the e.s.d. in the dihedral angle between two l.s. planes) are estimated using the full covariance matrix. The cell e.s.d.'s are taken into account individually in the estimation of e.s.d.'s in distances, angles and torsion angles; correlations between e.s.d.'s in cell parameters are only used when they are defined by crystal symmetry. An approximate (isotropic) treatment of cell e.s.d.'s is used for estimating e.s.d.'s involving l.s. planes.
Refinement. Refinement of *F*^2^ against ALL reflections. The weighted *R*-factor *wR* and goodness of fit *S* are based on *F*^2^, conventional *R*-factors *R* are based on *F*, with *F* set to zero for negative *F*^2^. The threshold expression of *F*^2^ > σ(*F*^2^) is used only for calculating *R*-factors(gt) *etc*. and is not relevant to the choice of reflections for refinement. *R*-factors based on *F*^2^ are statistically about twice as large as those based on *F*, and *R*- factors based on ALL data will be even larger.

### Fractional atomic coordinates and isotropic or equivalent isotropic displacement parameters (Å^2^)

**Table:**

	*x*	*y*	*z*	*U*_iso_*/*U*_eq_	
O1	0.16074 (8)	0.41514 (14)	0.53315 (7)	0.0317 (3)	
O2	0.02540 (12)	1.06889 (18)	0.61544 (8)	0.0608 (5)	
O3	0.15045 (11)	1.04404 (18)	0.70707 (8)	0.0506 (4)	
N1	0.23549 (10)	0.23375 (17)	0.47826 (8)	0.0264 (3)	
N2	0.27422 (10)	0.41366 (17)	0.39899 (8)	0.0280 (3)	
N3	0.31349 (10)	0.47425 (16)	0.52541 (8)	0.0257 (3)	
N4	0.41782 (10)	0.47197 (17)	0.64801 (8)	0.0278 (4)	
N5	0.30600 (10)	0.26475 (17)	0.60546 (8)	0.0264 (3)	
N6	0.26077 (10)	0.00320 (18)	0.55444 (8)	0.0295 (4)	
N7	0.09529 (12)	0.99495 (19)	0.64927 (9)	0.0365 (4)	
C1	0.23052 (12)	0.0710 (2)	0.48797 (10)	0.0260 (4)	
C2	0.21032 (12)	0.0005 (2)	0.41596 (10)	0.0274 (4)	
C3	0.19231 (12)	−0.1576 (2)	0.39047 (10)	0.0307 (4)	
H3A	0.1886	−0.2423	0.4221	0.037*	
C4	0.18000 (13)	−0.1875 (2)	0.31828 (11)	0.0339 (5)	
H4A	0.1664	−0.2940	0.2999	0.041*	
C5	0.18705 (14)	−0.0644 (2)	0.27145 (11)	0.0349 (5)	
H5A	0.1789	−0.0893	0.2221	0.042*	
C6	0.20568 (12)	0.0928 (2)	0.29572 (10)	0.0314 (4)	
H6A	0.2113	0.1756	0.2639	0.038*	
C7	0.21605 (12)	0.1264 (2)	0.36818 (10)	0.0279 (4)	
C8	0.23789 (12)	0.2738 (2)	0.41126 (10)	0.0267 (4)	
C9	0.31727 (12)	0.5053 (2)	0.45789 (10)	0.0268 (4)	
C10	0.38989 (12)	0.6226 (2)	0.46887 (10)	0.0285 (4)	
C11	0.42434 (13)	0.6997 (2)	0.42060 (11)	0.0326 (4)	
H11A	0.3973	0.6845	0.3694	0.039*	
C12	0.49891 (14)	0.7987 (2)	0.44965 (11)	0.0349 (5)	
H12A	0.5225	0.8544	0.4176	0.042*	
C13	0.54074 (13)	0.8193 (2)	0.52464 (11)	0.0331 (5)	
H13A	0.5921	0.8885	0.5425	0.040*	
C14	0.50902 (12)	0.7412 (2)	0.57373 (10)	0.0308 (4)	
H14A	0.5383	0.7540	0.6249	0.037*	
C15	0.43269 (12)	0.6432 (2)	0.54535 (10)	0.0283 (4)	
C16	0.38688 (12)	0.5356 (2)	0.58077 (10)	0.0276 (4)	
C17	0.38092 (12)	0.3310 (2)	0.65744 (9)	0.0268 (4)	
C18	0.42053 (12)	0.2038 (2)	0.70988 (9)	0.0274 (4)	
C19	0.49496 (12)	0.2021 (2)	0.77467 (10)	0.0317 (4)	
H19A	0.5284	0.2977	0.7932	0.038*	
C20	0.51877 (13)	0.0575 (2)	0.81119 (11)	0.0384 (5)	
H20A	0.5679	0.0548	0.8565	0.046*	
C21	0.47226 (13)	−0.0857 (2)	0.78312 (11)	0.0384 (5)	
H21A	0.4902	−0.1832	0.8097	0.046*	
C22	0.40048 (13)	−0.0871 (2)	0.71720 (10)	0.0341 (4)	
H22A	0.3709	−0.1850	0.6971	0.041*	
C23	0.37281 (12)	0.0595 (2)	0.68102 (10)	0.0282 (4)	
C24	0.30390 (12)	0.0996 (2)	0.61117 (10)	0.0275 (4)	
C25	0.14725 (12)	0.5556 (2)	0.56414 (10)	0.0273 (4)	
C26	0.19917 (12)	0.6002 (2)	0.63496 (10)	0.0307 (4)	
H26A	0.2461	0.5311	0.6640	0.037*	
C27	0.18261 (12)	0.7450 (2)	0.66318 (10)	0.0302 (4)	
H27A	0.2188	0.7774	0.7111	0.036*	
C28	0.11247 (13)	0.8418 (2)	0.62041 (10)	0.0290 (4)	
C29	0.05805 (13)	0.7966 (2)	0.55091 (10)	0.0322 (4)	
H29A	0.0088	0.8630	0.5232	0.039*	
C30	0.07611 (12)	0.6538 (2)	0.52219 (10)	0.0299 (4)	
H30A	0.0402	0.6226	0.4740	0.036*	
B1	0.24932 (14)	0.3530 (2)	0.53782 (11)	0.0264 (4)	

### Atomic displacement parameters (Å^2^)

**Table:**

	*U*^11^	*U*^22^	*U*^33^	*U*^12^	*U*^13^	*U*^23^
O1	0.0254 (7)	0.0321 (7)	0.0366 (8)	0.0012 (5)	0.0094 (6)	−0.0069 (6)
O2	0.0690 (12)	0.0495 (9)	0.0467 (10)	0.0283 (8)	−0.0017 (9)	−0.0077 (7)
O3	0.0539 (10)	0.0491 (9)	0.0383 (9)	0.0037 (7)	0.0027 (8)	−0.0168 (7)
N1	0.0260 (8)	0.0272 (8)	0.0251 (8)	0.0008 (6)	0.0077 (6)	0.0006 (6)
N2	0.0279 (8)	0.0262 (8)	0.0267 (8)	0.0025 (6)	0.0054 (7)	0.0022 (6)
N3	0.0253 (8)	0.0254 (7)	0.0246 (8)	0.0018 (6)	0.0065 (6)	−0.0008 (6)
N4	0.0277 (8)	0.0296 (8)	0.0261 (9)	0.0004 (6)	0.0091 (7)	−0.0016 (6)
N5	0.0250 (8)	0.0296 (8)	0.0246 (8)	0.0006 (6)	0.0088 (6)	0.0011 (6)
N6	0.0261 (8)	0.0330 (8)	0.0297 (9)	−0.0038 (6)	0.0099 (7)	0.0015 (7)
N7	0.0424 (10)	0.0367 (9)	0.0281 (9)	0.0037 (8)	0.0092 (8)	−0.0025 (7)
C1	0.0226 (9)	0.0270 (9)	0.0283 (10)	−0.0004 (7)	0.0089 (8)	0.0015 (7)
C2	0.0221 (9)	0.0315 (9)	0.0272 (10)	0.0012 (7)	0.0069 (8)	−0.0008 (7)
C3	0.0276 (10)	0.0292 (9)	0.0348 (11)	−0.0003 (7)	0.0102 (8)	−0.0017 (8)
C4	0.0315 (11)	0.0316 (10)	0.0377 (12)	−0.0021 (8)	0.0111 (9)	−0.0075 (8)
C5	0.0362 (11)	0.0378 (11)	0.0300 (11)	0.0006 (8)	0.0108 (9)	−0.0065 (8)
C6	0.0297 (10)	0.0355 (10)	0.0283 (10)	0.0004 (8)	0.0092 (8)	0.0012 (8)
C7	0.0223 (9)	0.0304 (9)	0.0289 (10)	0.0003 (7)	0.0061 (8)	−0.0015 (7)
C8	0.0233 (9)	0.0299 (9)	0.0257 (10)	0.0034 (7)	0.0068 (7)	0.0018 (7)
C9	0.0281 (10)	0.0253 (9)	0.0257 (10)	0.0046 (7)	0.0078 (8)	0.0025 (7)
C10	0.0281 (10)	0.0241 (9)	0.0326 (11)	0.0047 (7)	0.0098 (8)	0.0009 (7)
C11	0.0362 (11)	0.0266 (9)	0.0360 (11)	0.0059 (8)	0.0138 (9)	0.0027 (8)
C12	0.0379 (11)	0.0272 (9)	0.0453 (13)	0.0038 (8)	0.0217 (10)	0.0032 (8)
C13	0.0266 (10)	0.0249 (9)	0.0487 (13)	0.0017 (7)	0.0143 (9)	−0.0002 (8)
C14	0.0269 (10)	0.0275 (9)	0.0340 (11)	0.0046 (7)	0.0054 (8)	0.0002 (8)
C15	0.0272 (10)	0.0241 (9)	0.0331 (11)	0.0036 (7)	0.0100 (8)	0.0017 (7)
C16	0.0260 (10)	0.0261 (9)	0.0294 (10)	0.0019 (7)	0.0079 (8)	−0.0033 (7)
C17	0.0226 (9)	0.0333 (10)	0.0244 (10)	0.0003 (7)	0.0078 (8)	−0.0025 (7)
C18	0.0255 (10)	0.0343 (10)	0.0244 (10)	0.0007 (7)	0.0113 (8)	0.0020 (7)
C19	0.0259 (10)	0.0403 (11)	0.0291 (10)	−0.0021 (8)	0.0098 (8)	−0.0005 (8)
C20	0.0278 (11)	0.0491 (12)	0.0335 (11)	−0.0006 (8)	0.0047 (9)	0.0086 (9)
C21	0.0299 (11)	0.0423 (11)	0.0404 (12)	0.0019 (8)	0.0089 (9)	0.0132 (9)
C22	0.0303 (11)	0.0374 (10)	0.0351 (11)	−0.0002 (8)	0.0121 (9)	0.0063 (8)
C23	0.0269 (10)	0.0340 (10)	0.0262 (10)	0.0001 (7)	0.0121 (8)	0.0035 (8)
C24	0.0268 (10)	0.0297 (9)	0.0285 (10)	−0.0015 (7)	0.0128 (8)	0.0015 (7)
C25	0.0259 (10)	0.0288 (9)	0.0293 (10)	−0.0010 (7)	0.0124 (8)	−0.0014 (7)
C26	0.0239 (10)	0.0403 (10)	0.0260 (10)	0.0039 (8)	0.0061 (8)	0.0000 (8)
C27	0.0261 (10)	0.0400 (10)	0.0247 (10)	−0.0016 (8)	0.0090 (8)	−0.0022 (8)
C28	0.0316 (10)	0.0306 (9)	0.0267 (10)	−0.0023 (7)	0.0125 (8)	−0.0019 (7)
C29	0.0331 (11)	0.0326 (10)	0.0273 (10)	0.0020 (8)	0.0061 (8)	0.0013 (8)
C30	0.0302 (10)	0.0330 (10)	0.0244 (10)	0.0001 (8)	0.0068 (8)	−0.0006 (8)
B1	0.0245 (11)	0.0280 (10)	0.0264 (11)	0.0012 (8)	0.0084 (9)	−0.0008 (8)

### Geometric parameters (Å, °)

**Table:**

O1—C25	1.363 (2)	C10—C11	1.394 (3)
O1—B1	1.453 (2)	C10—C15	1.420 (3)
O2—N7	1.228 (2)	C11—C12	1.378 (3)
O3—N7	1.229 (2)	C11—H11A	0.9500
N1—C8	1.364 (2)	C12—C13	1.393 (3)
N1—C1	1.369 (2)	C12—H12A	0.9500
N1—B1	1.485 (2)	C13—C14	1.385 (3)
N2—C9	1.349 (2)	C13—H13A	0.9500
N2—C8	1.350 (2)	C14—C15	1.392 (3)
N3—C9	1.365 (2)	C14—H14A	0.9500
N3—C16	1.374 (2)	C15—C16	1.461 (3)
N3—B1	1.500 (2)	C17—C18	1.451 (2)
N4—C16	1.341 (2)	C18—C19	1.392 (3)
N4—C17	1.345 (2)	C18—C23	1.421 (3)
N5—C17	1.372 (2)	C19—C20	1.379 (3)
N5—C24	1.376 (2)	C19—H19A	0.9500
N5—B1	1.502 (2)	C20—C21	1.401 (3)
N6—C1	1.342 (2)	C20—H20A	0.9500
N6—C24	1.344 (2)	C21—C22	1.385 (3)
N7—C28	1.453 (2)	C21—H21A	0.9500
C1—C2	1.454 (2)	C22—C23	1.398 (3)
C2—C3	1.397 (2)	C22—H22A	0.9500
C2—C7	1.424 (2)	C23—C24	1.455 (3)
C3—C4	1.378 (3)	C25—C26	1.390 (2)
C3—H3A	0.9500	C25—C30	1.394 (2)
C4—C5	1.401 (3)	C26—C27	1.383 (3)
C4—H4A	0.9500	C26—H26A	0.9500
C5—C6	1.383 (3)	C27—C28	1.383 (3)
C5—H5A	0.9500	C27—H27A	0.9500
C6—C7	1.396 (2)	C28—C29	1.381 (3)
C6—H6A	0.9500	C29—C30	1.382 (2)
C7—C8	1.457 (2)	C29—H29A	0.9500
C9—C10	1.455 (3)	C30—H30A	0.9500
			
C25—O1—B1	124.58 (14)	C13—C14—H14A	121.2
C8—N1—C1	113.27 (14)	C15—C14—H14A	121.2
C8—N1—B1	123.03 (15)	C14—C15—C10	121.07 (17)
C1—N1—B1	123.23 (15)	C14—C15—C16	131.53 (17)
C9—N2—C8	116.73 (15)	C10—C15—C16	107.15 (15)
C9—N3—C16	112.71 (15)	N4—C16—N3	123.03 (16)
C9—N3—B1	122.61 (15)	N4—C16—C15	129.40 (16)
C16—N3—B1	123.05 (15)	N3—C16—C15	105.50 (15)
C16—N4—C17	116.75 (15)	N4—C17—N5	122.97 (16)
C17—N5—C24	112.15 (15)	N4—C17—C18	129.05 (16)
C17—N5—B1	123.28 (15)	N5—C17—C18	106.13 (15)
C24—N5—B1	122.21 (15)	C19—C18—C23	120.77 (16)
C1—N6—C24	117.15 (15)	C19—C18—C17	131.87 (16)
O2—N7—O3	122.68 (17)	C23—C18—C17	107.18 (15)
O2—N7—C28	118.57 (16)	C20—C19—C18	118.05 (17)
O3—N7—C28	118.74 (16)	C20—C19—H19A	121.0
N6—C1—N1	121.98 (16)	C18—C19—H19A	121.0
N6—C1—C2	130.79 (16)	C19—C20—C21	121.60 (19)
N1—C1—C2	105.44 (15)	C19—C20—H20A	119.2
C3—C2—C7	120.36 (17)	C21—C20—H20A	119.2
C3—C2—C1	132.24 (17)	C22—C21—C20	121.01 (18)
C7—C2—C1	107.32 (15)	C22—C21—H21A	119.5
C4—C3—C2	118.25 (17)	C20—C21—H21A	119.5
C4—C3—H3A	120.9	C21—C22—C23	118.17 (18)
C2—C3—H3A	120.9	C21—C22—H22A	120.9
C3—C4—C5	121.49 (17)	C23—C22—H22A	120.9
C3—C4—H4A	119.3	C22—C23—C18	120.29 (17)
C5—C4—H4A	119.3	C22—C23—C24	132.28 (17)
C6—C5—C4	121.21 (18)	C18—C23—C24	107.23 (15)
C6—C5—H5A	119.4	N6—C24—N5	123.04 (16)
C4—C5—H5A	119.4	N6—C24—C23	129.39 (16)
C5—C6—C7	118.18 (17)	N5—C24—C23	105.86 (15)
C5—C6—H6A	120.9	O1—C25—C26	122.87 (16)
C7—C6—H6A	120.9	O1—C25—C30	116.98 (16)
C6—C7—C2	120.47 (16)	C26—C25—C30	120.12 (16)
C6—C7—C8	132.45 (17)	C27—C26—C25	120.16 (17)
C2—C7—C8	106.99 (15)	C27—C26—H26A	119.9
N2—C8—N1	122.25 (16)	C25—C26—H26A	119.9
N2—C8—C7	130.40 (16)	C28—C27—C26	118.85 (17)
N1—C8—C7	105.73 (14)	C28—C27—H27A	120.6
N2—C9—N3	122.80 (16)	C26—C27—H27A	120.6
N2—C9—C10	129.53 (17)	C29—C28—C27	121.77 (17)
N3—C9—C10	106.11 (15)	C29—C28—N7	118.99 (16)
C11—C10—C15	120.32 (17)	C27—C28—N7	119.24 (16)
C11—C10—C9	132.33 (17)	C28—C29—C30	119.24 (17)
C15—C10—C9	107.16 (15)	C28—C29—H29A	120.4
C12—C11—C10	117.79 (18)	C30—C29—H29A	120.4
C12—C11—H11A	121.1	C29—C30—C25	119.78 (17)
C10—C11—H11A	121.1	C29—C30—H30A	120.1
C11—C12—C13	121.91 (18)	C25—C30—H30A	120.1
C11—C12—H12A	119.0	O1—B1—N1	108.11 (15)
C13—C12—H12A	119.0	O1—B1—N3	115.48 (15)
C14—C13—C12	121.35 (18)	N1—B1—N3	104.11 (14)
C14—C13—H13A	119.3	O1—B1—N5	119.21 (15)
C12—C13—H13A	119.3	N1—B1—N5	104.25 (14)
C13—C14—C15	117.54 (17)	N3—B1—N5	104.16 (14)
